# PLBD: protein–ligand binding database of thermodynamic and kinetic intrinsic parameters

**DOI:** 10.1093/database/baad040

**Published:** 2023-06-08

**Authors:** Darius Lingė, Marius Gedgaudas, Andrius Merkys, Vytautas Petrauskas, Antanas Vaitkus, Algirdas Grybauskas, Vaida Paketurytė, Asta Zubrienė, Audrius Zakšauskas, Aurelija Mickevičiūtė, Joana Smirnovienė, Lina Baranauskienė, Edita Čapkauskaitė, Virginija Dudutienė, Ernestas Urniežius, Aleksandras Konovalovas, Egidijus Kazlauskas, Kirill Shubin, Helgi B Schiöth, Wen-Yih Chen, John E Ladbury, Saulius Gražulis, Daumantas Matulis

**Affiliations:** Department of Biothermodynamics and Drug Design, Institute of Biotechnology, Life Sciences Center, Vilnius University, Saulėtekio 7, Vilnius LT-10257, Lithuania; Department of Biothermodynamics and Drug Design, Institute of Biotechnology, Life Sciences Center, Vilnius University, Saulėtekio 7, Vilnius LT-10257, Lithuania; Sector of Crystallography and Cheminformatics, Institute of Biotechnology, Life Sciences Center, Vilnius University, Saulėtekio 7, Vilnius LT-10257, Lithuania; Department of Biothermodynamics and Drug Design, Institute of Biotechnology, Life Sciences Center, Vilnius University, Saulėtekio 7, Vilnius LT-10257, Lithuania; Sector of Crystallography and Cheminformatics, Institute of Biotechnology, Life Sciences Center, Vilnius University, Saulėtekio 7, Vilnius LT-10257, Lithuania; Sector of Crystallography and Cheminformatics, Institute of Biotechnology, Life Sciences Center, Vilnius University, Saulėtekio 7, Vilnius LT-10257, Lithuania; Department of Biothermodynamics and Drug Design, Institute of Biotechnology, Life Sciences Center, Vilnius University, Saulėtekio 7, Vilnius LT-10257, Lithuania; Department of Biothermodynamics and Drug Design, Institute of Biotechnology, Life Sciences Center, Vilnius University, Saulėtekio 7, Vilnius LT-10257, Lithuania; Department of Biothermodynamics and Drug Design, Institute of Biotechnology, Life Sciences Center, Vilnius University, Saulėtekio 7, Vilnius LT-10257, Lithuania; Department of Biothermodynamics and Drug Design, Institute of Biotechnology, Life Sciences Center, Vilnius University, Saulėtekio 7, Vilnius LT-10257, Lithuania; Department of Biothermodynamics and Drug Design, Institute of Biotechnology, Life Sciences Center, Vilnius University, Saulėtekio 7, Vilnius LT-10257, Lithuania; Department of Biothermodynamics and Drug Design, Institute of Biotechnology, Life Sciences Center, Vilnius University, Saulėtekio 7, Vilnius LT-10257, Lithuania; Department of Biothermodynamics and Drug Design, Institute of Biotechnology, Life Sciences Center, Vilnius University, Saulėtekio 7, Vilnius LT-10257, Lithuania; Department of Biothermodynamics and Drug Design, Institute of Biotechnology, Life Sciences Center, Vilnius University, Saulėtekio 7, Vilnius LT-10257, Lithuania; Department of Biothermodynamics and Drug Design, Institute of Biotechnology, Life Sciences Center, Vilnius University, Saulėtekio 7, Vilnius LT-10257, Lithuania; Department of Biochemistry and Molecular Biology, Institute of Biosciences, Life Sciences Center, Vilnius University, Saulėtekio 7, Vilnius LT-10257, Lithuania; Department of Biothermodynamics and Drug Design, Institute of Biotechnology, Life Sciences Center, Vilnius University, Saulėtekio 7, Vilnius LT-10257, Lithuania; Latvian Institute of Organic Synthesis, Aizkraukles Street 21, Riga LV-1006, Latvia; Functional Pharmacology and Neuroscience, Department of Surgical Sciences, Uppsala University, Kirurgiska Vetenskaper, Box 593, Uppsala 751 24, Sweden; Department of Chemical and Materials Engineering, National Central University, No. 300, Zhongda Rd., Zhongli Dist., Taoyuan City, Jhong-Li 320, Taiwan; School of Molecular and Cellular Biology, University of Leeds, Leeds LS2 9JT, United Kingdom; Sector of Crystallography and Cheminformatics, Institute of Biotechnology, Life Sciences Center, Vilnius University, Saulėtekio 7, Vilnius LT-10257, Lithuania; Department of Biothermodynamics and Drug Design, Institute of Biotechnology, Life Sciences Center, Vilnius University, Saulėtekio 7, Vilnius LT-10257, Lithuania

## Abstract

We introduce a protein–ligand binding database (PLBD) that presents thermodynamic and kinetic data of reversible protein interactions with small molecule compounds. The manually curated binding data are linked to protein–ligand crystal structures, enabling structure–thermodynamics correlations to be determined. The database contains over 5500 binding datasets of 556 sulfonamide compound interactions with the 12 catalytically active human carbonic anhydrase isozymes defined by fluorescent thermal shift assay, isothermal titration calorimetry, inhibition of enzymatic activity and surface plasmon resonance. In the PLBD, the intrinsic thermodynamic parameters of interactions are provided, which account for the binding-linked protonation reactions. In addition to the protein–ligand binding affinities, the database provides calorimetrically measured binding enthalpies, providing additional mechanistic understanding. The PLBD can be applied to investigations of protein–ligand recognition and could be integrated into small molecule drug design.

**Database URL** https://plbd.org/

## Introduction

Protein**–**ligand interaction affinity data have been gathered in several public databases (1–11). The accumulation of thermodynamic and kinetic data in these databases deepens our knowledge of still poorly understood protein small molecule recognition and hence can be of value in drug development (12). Some are based on data mining from the RCSB Protein Data Bank (PDB) (13), with the aim of revealing structural aspects of binding. Protein–ligand binding databases (PLBDs) remain the primary source of information for computational and medicinal chemists designing molecules for new drugs (14, 15). However, current databases do not systematically address binding-linked reactions such as the protonation of the ligand or protein upon binding.

**Figure 1.**
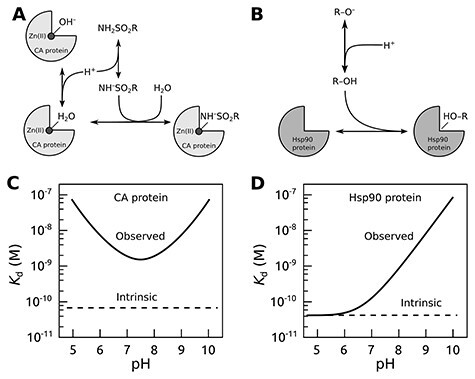
The concept of intrinsic binding parameters after accounting for ligand binding–linked protonation reactions. Upper panels show interactions occurring when carbonic anhydrase (CA; A) or Hsp90 (B) proteins bind sulfonamide or resorcinol-based ligands, respectively. The lower panels plot typical observed and intrinsic dissociation constants (affinities) as a function of pH. For the CA protein (C), it is usually impossible to find conditions where the observed and intrinsic values would coincide (17), while for Hsp90 ligands bearing the resorcinol group (D), the match was observed only at acidic pH (20, 21).

The affinity of a protein–ligand interaction may have significant contributions from binding-linked protonation interactions that in most cases cannot be ignored (16, 17). When subtracting these contributions, the remaining ‘true’ protein–ligand interaction parameters are called intrinsic (16–19). The linked interactions may strongly affect the binding parameters, and the intrinsic affinities can be calculated only with a detailed understanding of the binding mechanism. In the absence of consideration of linked interactions, data from different experiments may not be comparable. Figure 1 shows two examples of linked interactions for carbonic anhydrase (CA) and heat shock protein 90 (Hsp90) upon binding their ligands.

Here we primarily report data for the family of human CA enzymes that catalyze the reversible hydration of CO_2_ to HCO_3_^−^ and acid protons and participate in numerous physiological processes, especially pH homeostasis and carbon metabolism. The human body contains 12 distinct CA isozymes differently expressed in all tissues and cell types. Isozymes CAI, CAII, CAIII, CAVII and CAXIII are cytosolic; isozymes CAIX, CAXII and CAXIV are transmembrane proteins, while CAIV is attached to the cell membrane; CAVA and CAVB are mitochondrial, while CAVI is secreted. The CAII isozyme has been used as a model protein in various biophysical studies for decades (22). All the catalytically active CA isozymes contain a Zn^II^ ion in their active sites and share the binding mechanism outlined in Figure 1A, except for CAVIII, CAX and CAXI, which are non-catalytic. The primary sulfonamide-bearing compounds are good ligands of CA proteins and form a coordination bond between the sulfonamide amino group and the Zn^II^. A large series of sulfonamide compounds possess high affinity for CA isozymes, inhibit their catalytic activity and have been used as pharmaceuticals to regulate various diseases. However, the catalytic sites of CA isozymes that bind sulfonamide ligands are structurally similar, and it is difficult to make compounds that would bind with high selectivity and specificity toward only one isozyme (23). Because of these challenges, we found this family of enzymes to be a suitable group of model proteins that could be used to investigate and highlight the principles of recognition and ligand binding specificity for target-based drug design.

When sulfonamide binds a CA isozyme, both the protein and the compound undergo binding-linked protonation reactions. At pH 7.0, sulfonamides predominantly exist in the electrostatically neutral protonated form NH_2_SO_2_R because their p*K*_a_’s are around 10. However, they bind to the CA only in a negatively charged deprotonated form NH^–^SO_2_R. Thus, at pH 7.0, only 0.1% of the compound is in the binding-ready form. The remaining fraction must undergo deprotonation to bind the CA. Therefore, when the binding is measured by any experimental technique, the summed reaction of sulfonamide deprotonation and its binding to CA is observed. It is possible to dissect the two reactions by measuring the interaction at various pH conditions and determine the intrinsic affinity by accounting for the linked protonation reactions both for the ligand and protein (17). The intrinsic affinity is independent of pH, while the observed affinity depends on pH, and it is of great value that the pH is specified in the database along with the experimental data. Computational predictions that are based on correlations between the chemical structure of compound–protein pair and their interaction energy could become more effective if intrinsic parameters were used instead of the observed ones.

The protein–ligand binding affinity is the main but not the only thermodynamic parameter useful in the drug design. In addition to affinity (binding/dissociation constant, the change upon binding of the standard Gibbs energy, ∆*G*_b_), additional information about the binding mechanism could be obtained from the changes in the enthalpy (∆*H*), entropy (∆*S*), heat capacity (∆*C*_p_) and possibly other parameters. For example, if combined with crystallography or nuclear magnetic resonance (NMR) data, the change in enthalpy upon protein–ligand binding could give valuable insights into intermolecular bonds that result in favorable interaction pathways (24). Furthermore, the kinetics of binding may be as important for describing the binding interaction as thermodynamics. The association and dissociation rates also provide information about the interaction. Sulfonamide’s association with CAs depends on linked protonation interactions, and the intrinsic kinetics should be determined (25). Only a few of the aforementioned public databases of protein–ligand interactions provide most of these parameters reflecting the underuse of potentially valuable information.

Here we introduce a PLBD available at https://plbd.org. The PLBD contains experimentally observed thermodynamic and kinetic parameters and evaluates the intrinsic binding parameters when the pH-dependent protein–ligand interaction data are provided. We started building this database by gathering experimental results of the sulfonamide compound binding to the CA family of enzymes. Broad spectra of sulfonamide compound–binding affinities for CAs could be achieved by substituting or attaching various chemical groups and forming aliphatic or aromatic sulfonamides. In this way, PLBD helps to reveal how the substituting chemical group of the compound or minor changes in the protein structure of one isozyme compared to another affect the binding thermodynamics and kinetics.

## Methods

### Experimental techniques

#### Protein preparation

Proteins were recombinantly prepared by cloning their human genes in plasmids, expressed in bacterial or mammalian cells, and purified by ion-exchange and/or affinity chromatography. Protein purity was checked by sodium dodecyl sulphate-polyacrylamide gel electrophoresis and identity by High-Resolution Mass Spectrometry (HRMS), confirming the Molecular weight to 1 Da precision (26, 27). The enzymatic activity of each CA isozyme was determined (17, 28, 29).

#### Compound synthesis

The chemical compounds were synthesized and purified, and their purity was confirmed by thin-layer chromatography and/or high pressure (or high performance) liquid chromatography and the identity by HRMS, by elemental analysis and by proton, carbon and, when available, fluorine NMR as previously described and reviewed (17, 30, 31).

#### X-ray crystallography

We have determined 105 X-ray crystallographic structures of several CA isozymes (CAI, CAII, CAIV, CAXII and CAXIII) in complex with bound inhibitors and deposited to the PDB (32). In the PLBD, each available protein–compound structure is associated with every performed thermodynamic and kinetic measurement of binding and with the calculated intrinsic binding parameters.

#### Binding energetics

Thermodynamic parameters of protein–compound interaction (dissociation constant, association constant, standard Gibbs energy and enthalpy of binding) were experimentally determined by one or more of the four experimental techniques: (i) thermal shift assay (TSA), (ii) isothermal titration calorimetry (ITC), (iii) stopped-flow assay (SFA) of the enzymatic activity inhibition and (iv) surface plasmon resonance (SPR). The kinetic parameters (the rates of association and dissociation) were determined by SPR.

#### Thermal shift assay

The TSA (also termed fluorescence-based TSA (FTSA), differential scanning fluorimetry (DSF)) is based on protein thermal stabilization by binding ligands (33, 34). The melting temperatures of a protein were determined at increasing concentrations of added ligand. Analysis of the experimental data provided the affinity of protein–ligand interaction (Gibbs energy of binding or dissociation constant) (35, 36). This high-throughput technique requires low amount of samples and can be performed on real-time polymerase chain reaction machines.

#### Isothermal titration calorimetry

ITC is one of the most commonly used techniques to study protein–ligand interaction (37). In addition to the binding affinity, it determines the changes in standard enthalpy upon protein–ligand binding.

#### Inhibition of enzymatic activity

Inhibition by the compounds was determined by the SFA that follows the change in pH of solution upon acidification when the CA hydrates carbon dioxide to bicarbonate anion and acid proton (28).

#### Kinetics of binding

The compound association and dissociation rates with each CA isozyme were determined by SPR using the Biacore T200 instrument as previously described (17, 38).

The advantages and limitations of ITC, TSA, SFA and SPR techniques in determining protein–ligand interaction parameters were discussed in greater detail by Linkuvienė *et al.* (17).

### Representation of datasets

The PLBD is intended to be a constantly evolving database, with new data added in real time as the measurements progress. Therefore, it is impossible to represent it as a static collection of files, like those published in, e.g. Zenodo or Data Dryad data repositories. Instead, we have chosen to publish the database as a ‘live’ server on the dedicated Web address (https://plbd.org/) and represent the PLBD datasets as resources on this server.

#### Database engine

The underlying Database Management System is implemented using an SQL (39) server. The SQL data description was chosen over other solutions, such as file-based approaches or NoSQL because it offers superb data integrity solutions and query capabilities. In particular, the SQL has a standardized language description (39), time-proven performance and maintenance compatibility between previous versions, mature engines (MySQL (40), PostgreSQL (41) and SQLite (42)), a rich set of data description features and a flexible query language. Modern SQL servers offer as a standard ACID (43) compliance, transactions, uniqueness and not null constraints, data format guaranties (typed data) and foreign key constraint guaranties—a collection of features that no current NoSQL systems are capable of. These features allow scientists to insert new data without compromising data integrity and database maintainers to curate data in the database without causing incompatibilities with previous database revisions and without breaking promises for database clients.

We designed the PLBD to map the experimental concepts directly into the SQL relational schema. Thus, we create a separate table for each type of experimental object, where each row represents a distinct object of that type. Unique keys maintained by SQL engines ensure that these objects can be unambiguously referenced.

#### Documentation of data semantics

The SQL alone cannot handle the full semantics of scientific data. Thus, we have designed an extensible markup language (XML) schema that formally specifies additional fields necessary to describe metadata presentation needs (44). All aspects of PLBD are described in an XML file that validates against the XML schema. Thus, all metadata are available in a single definitive description. From this XML file, SQL data definition statements are generated using converter software (45), as well as metadata necessary to represent data as Web documents or structured data streams in JSON, XML or CSV. Translation between the SQL database and various streamed formats is performed by a middleware layer RestfulDB (46). To ensure smooth co-development of the XML schema, the XML database description document and the conversion software, Semantic Versioning (SemVer (47)) is employed for both the schema and the XML document.

The SQL databases use primary keys as integer identifiers (IDs), while foreign key constraints maintain the integrity of the data table connections. However, these integer keys do not always serve as stable external identifiers because they sometimes must be changed, e.g. in case of data curation or when two separate databases of similar structure are merged. Global identifier schemas such as ARK, Handle or DOI are too complex and costly to maintain for PLBD. Thus, we used labels and universally unique identifiers (UUIDs) (48) as stable external identifiers. The database engine enforces the uniqueness of UUIDs within the database table. Labels, which are supposed to be human-usable identifiers, are assigned by the data depositor with suggestions by the RestfulDB. Although UUIDs are much less human-readable, they can be generated offline without consulting the existing database tables, and the underlying generation algorithms ensure their uniqueness (48). Both labels and UUIDs can be used to identify database records and construct unique stable URLs to access those records via the Web. The database schemas are available at the PLBD server at https://plbd.org/doc/db/schemas and as a Zenodo dataset (44).

#### Reproducible queries

Stable external identifiers are necessary but insufficient to ensure reproducible queries in the scientific databases. The PLBD can trace the changes in data because each database record has a revision ID field. This field gets a unique value with each database change or related group of changes (insert, delete or update operations). Revision IDs are incremental; records with larger revision IDs appear later. A dedicated revision tablecontains descriptions of all revisions, registering who, when and why made the change. When data are updated or deleted, old values need to be retained to enable queries of the previous revisions. In the PLBD, we achieved this functionality by creating history tables for each data table. If data are modified or deleted, a dedicated SQL trigger copies old values from the main data table to the corresponding history table. The revision in which data were modified or deleted is also inserted into the history table. Thus, history tables enable querying those values in the database at any revision.

#### Representation on the World Wide Web

Modern scientific databases should be accessible via the Web interface. In PLBD, we would like to provide our data browsable for humans as a set of HTML pages and also ready for reuse by other software. To meet the latter requirement, we present our database via a REST-style interface (49). Since the SQL database engines in PLBD do not support the REST interface out of the box, we have developed a middleware layer, called RestfulDB, that can be targeted to any SQL database (currently supported engines are MySQL, SQLite2 and SQLite3), and the database tables, together with their links over foreign keys, can be presented via an HTTP connection using REST. The same RestfulDB middleware layer can represent SQL tables as HTML pages for browsing. The RestfulDB is currently implemented in Perl (50) programming language. Additional supported data download formats are CSV (51, 52), ODT (ODS), XLS and XLSX. If the table structure changes, the user-accessible REST interface will also change. We version the database schema using Semantic Versioning (47) conventions to enable reproducible queries and smooth interaction with REST clients. Small changes such as a column title (without changing programmatic column names) and improving descriptions or documentation will be indicated as patch revisions of the SemVer. Adding tables and columns yields minor version changes, while changing the column names and types, deleting the columns, reorganizing tables and changing measurement units will cause major (incompatible) version changes. PLBD stores the current version and the version change history in a special version table. Thus, a client can detect programmatically whether it is compatible with the PLBD instance in use.

Using the SQL engine enables us to present information as ‘bare’ data tables and as datasets of preselected or computed values. Such presentations are possible using SQL views. Thus, the most reliable measurement data are selected and averaged, and intrinsic thermodynamic constants are automatically computed from the actual experimental values in the SQL view intrinsic parameters. The formulas used to compute the intrinsic values, their SQL implementation and the references to the original publications where these formulae are derived are given in a machine-readable form in the database description XML file. The SQL implementation is transferred to the working SQL database schema using the automatic conversion scripts (53), thus ensuring the single source of information for all PLBD instances. In this way, all mathematical procedures used to calculate the derived parameters (such as intrinsic thermodynamic parameters) are traceable to their original measurements and calculations for humans and software tools. One can also formally verify that the SQL implementation calculates the formula provided in the XML file; for such automatic verification, however, the tools do not exist; therefore, the check must be done manually. Other views offer joins between protein and compound batches and the actual compound with their properties, thus saving a user the need to perform table joins. The main database is also implemented as a set of SQL views and offers access to published data for everyone without registering an account. For a user, the views can be used the same way as regular tables, including access via the RestfulDB layer, except that the views can only be read and not updated. Since an anonymous user is not authorized to edit data, it is deemed that exposing views along with the regular tables will be a convenience.

## Results

**Figure 2.**
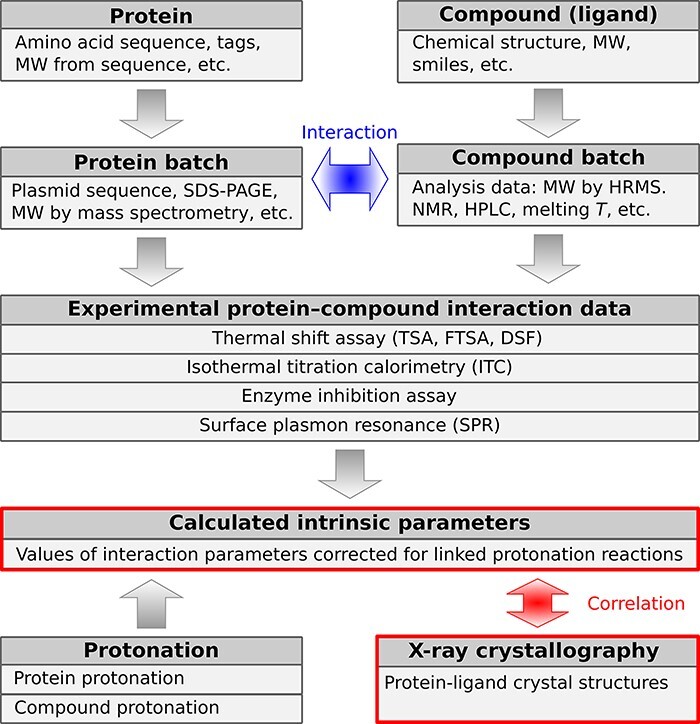
A schematic representation of PLBD. The database lists information about the structure, purification, actual batches of proteins and compounds and thermodynamic and structural information on protein–compound interaction. Arrows indicate the direction of information exchange between major structural units of the PLBD. The database structure allows users to trace all relationships between the deposited data.

Structurally, the PLBD is composed of data tables (samples, experiments and others) and views. The views display calculated parameters and other information derived from the primary data tables. Figure 2 shows a simplified schematic representation of the major PLBD structural data units of the database. Arrows in the scheme indicate the direction of information exchange between structural data units.

The first group of tables describes the chemical compounds and recombinant proteins. Information about proteins and compounds is listed in separate tables from their corresponding batches because each compound or protein may have been produced several times with potentially distinct yields or purities and different analytical techniques may have been used to characterize each batch. The experimental tables are related to batch tables in a many-to-one manner, and batch tables are also in many-to-one relation with protein and compound tables, meaning that each binding experiment will be related to a single batch of protein and ligand. The next group of tables contains the thermodynamic and kinetic data of protein interaction with chemical compounds (ligands). Experimentally measured parameters are listed in separate tables based on the technique used to determine a particular interaction: TSA, ITC, SFA and SPR. Many interaction datasets are complemented with the structural protein–ligand information from the PDB. Special attention in the PLBD is dedicated to the table of calculated intrinsic parameters that can be determined if both the protein and compound protonation information is available. For this purpose, the database also contains tables of protein and ligand protonation parameters (p*K*_a_ and ∆*H*_a_).

The experimental data tables have supplementary relationships, providing metadata of a particular experiment: who performed the assay, a device and the raw data/analysis files, a reference to the primary publication of the obtained results, etc. Such an approach enables us to trace the binding parameters from the original measurement through sample preparation, data acquisition and processing workflow.

Currently, the PLBD contains over 5500 binding parameters for more than 30 proteins and 580 compounds. For those protein–ligand interactions that have been measured by ITC, the standard changes in binding enthalpy are provided in addition to their affinities. Approximately, 130 X-ray crystallographic structures supplement the thermodynamic information of protein–ligand interaction. Most PLBD data are related to binding parameters of sulfonamide compounds’ interaction with 12 catalytically active human CA isozymes. Not all sulfonamide compound affinities have been determined for every CA isozyme. Over 2000 intrinsic binding parameters are available for various CA isozymes and their ligands. The PLBD also contains binding affinities of the Hsp90 and some of its ligands. The TSA and ITC data are available on two well-established Hsp90 inhibitors—17-AAG and radicicol (20, 21). The PLBD currently contains TSA and ITC results for 18 compounds binding to recombinant N-terminal domains of the Hsp90α and Hsp90β isozymes.

As an example of the database’s usefulness, we searched for compounds with the highest observed affinity (*K*_d,obs_) for each CA isozyme (Table 1). The corresponding intrinsic affinities (*K*_d,int_) are also listed. Note that there is only a limited correlation between the observed and intrinsic affinities. We pay special attention to the accuracy and precision of the experimental data. Selected reactions have been repeated many times, and the standard error and deviation of the data were estimated (54, 55). The current major limitation of the PLBD is that only several protein families are included in the database.

**Table 1.** List of compounds that exhibited the highest observed affinity for each CA isozyme as determined by TSA, selected from the entire list of compounds in the PLBD

Isozyme	Compound	*K*_d,obs_ (nM)	*K*_d,int_ (nM)
CAI	VD11-61	0.025	0.00066
CAII	VD10-49	0.79	0.045
CAIII	TFMSA; TFS	1000	220
CAIV	EA3-2	1.4	0.0042
CAVA	EZA	19	1.7
CAVB	VD12-05	0.050	0.0017
CAVI	TFMSA; TFS	14	1.2
CAVII	VD10-49	0.22	0.011
CAIX	VD11-4-2	0.083	0.00078
CAXII	EA12-3	0.40	0.0015
CAXIII	VD11-9	0.28	0.021
CAXIV	VD10-49	0.50	0.025

The corresponding intrinsic affinities calculated as described in (17) are also listed for each compound–protein pair. All values are determined at 37°C.

The FAIR data principles provide guidelines for how scientific data should be managed and reused after the data publication (56). To achieve this goal, one must first describe data structure and semantics. Relational data model can be viewed as a most general way to describe experimental scientific data (57–59). This model was originally developed for exchanging crystallographic data in the CIF framework (60, 61) but later adapted to other areas of science (62). The database schema and deposited data have revision and versioning systems providing historical traces of its evolution. We expect the synergy between an extensive dataset of protein–ligand thermodynamic parameters, and a comprehensive data storage and manipulation engine could deepen our understanding of the protein–ligand recognition principles.

## Technical notes

### Data integrity checks

The input stage of the dataset was validated using SQL constraints and SQL type system. Descriptions of the tables contain the following data constraints that guarantee the integrity of the data:

‧ SQL data type declarations. Numeric data types are declared as either INTEGER or FLOAT, as appropriate for the corresponding column type. The underlying SQL engine guarantees that the data are of the appropriate type; one cannot insert incorrect data into the table.

‧ UNIQUE constraints are used to ensure that record identifiers are not duplicated.

‧ FOREIGN KEY constraints are endorsed in the underlying SQL engine to ensure that connections between tables are not broken when data are modified.

‧ NOT NULL constraints denote obligatory data fields.

The SQL constraints ensure certain declared invariants on the provided data on which all database clients and users can rely. In particular, external data identifiers are guaranteed to be unique and can be used to identify specific data records. Types and uniqueness constraints guarantee the uniformity of the data.

For large uploaded data files, data integrity cannot be generally verified using SQL alone. For such data items, cryptographic checksums (e.g. SHA256 and MD5) are stored in the database tables. The checksums can be calculated automatically in the upload process or provided by the client. Data integrity checks can then be performed offline using external tools.

Certain aspects of the data cannot be expressed in SQL alone. For example, SQL does not provide means to specify units, relate checksums to the uploaded data files, specify MIME types of uploaded data, etc. These additional data constraints are described in an XML file. The XML file also contains necessary information to create an SQL schema (such as SQL data types and constraints), ensuring that SQL descriptions are consistent with the XML description. The SQL statements with the SQL database schema (i.e. the CREATE TABLE and CREATE VIEW statements) are then generated automatically from this single description with the help of the dedicated open-source converter xml2sqlschema (45), thus ensuring a single point of documentation for the database description. The XML description file itself conforms and is validated against the XML schema published online on the PLBD server (http://crystallography.net/xml/schema/relational-database-restfuldb/) and as a file archive (44).

### Usage suggestions

The PLBD can be used in three different ways. First, users can browse the data tables using a regular Web browser at the URL https://plbd.org. The HTML pages are generated on the fly from the underlying SQL tables by the RestfulDB middleware. Second, for programmatic access, the same RestfulDB software accepts standard HTTP GET, POST, PUT, PATCH and DELETE requests and can yield responses in standard HTML, CSV and JSON formats. The REST interface details are described in the RestfulDB design description, available in the main project repository (46), as release snapshot (63) and online (https://saulius.grazulis.lt/restfuldb/tags/v0.16.0/doc/converted/RESTful-script-design.pdf). Available REST end points correspond to the database itself, database tables (i.e. have the same names as database tables) and individual database records, identified by any unique database field value. Third, the data tables can be downloaded from the interactive Web pages in standardized CSV (51, 52), ODS, XLS and XLSX formats.

Related database tables are represented in tabular formats as multiple tabs (ODS and XLS(X) formats), as multiple CSV files packed in a ZIP format (CSV + ZIP format) or as external relations in JSON (64, 65) that follow JSON API (66) recommendations.

### Code availability

The restfuldb middleware code is available under GPL v2 or higher at svn://saulius-grazulis.lt/restfuldb. The released version of the code used for this manuscript is deposited to Zenodo (63). The SQL schema generation tools are available under GPL v3 or higher at svn://www.crystallography.net/solsa-database-scripts. The released version of the code used for this manuscript is deposited to Zenodo (45). Dataset XML description files are available at https://plbd.org/doc/db/schemas, and the XML schemas used for their validation and documenting the semantics of the XML data elements used in the database descriptions is available at https://plbd.org/doc/xml/schemas/relational-database-restfuldb. The current version of the XML, the generated SQL files and the associated XML schemas are deposited to Zenodo (44).

## Data Availability

Links to the data are provided in the manuscript. Snapshots of data and code are deposited to Zenodo (44, 45, 63).
